# Herbal Therapies and Social-Health Policies: Indigenous Ati Negrito Women's Dilemma and Reproductive Healthcare Transitions in the Philippines

**DOI:** 10.1155/2015/491209

**Published:** 2015-08-04

**Authors:** Homervergel G. Ong, Young-Dong Kim

**Affiliations:** Department of Life Science, Hallym University, 8310 Life Science Building, Hallymdaehak-Gil 1, Chuncheon City 200-702, Republic of Korea

## Abstract

The high maternal mortality in the Philippines in the past decades prompted intervention strategies to curb unwanted deaths of mothers and improve health and social conditions of women. Such introductions however have begun to challenge traditional reproductive health practices creating confusion among practitioners and incipient transitions in healthcare. Our aim in this study was to document the herbal therapies practiced by indigenous Ati Negrito women and discuss the implications of social and conventional healthcare intervention programs on reproductive healthcare traditions by conducting semistructured interviews. Fidelity Level index was used to determine culturally important plants (i.e., the most preferred). Review of related studies on most preferred plants and therapies was further carried out to provide information regarding their safety/efficacy (or otherwise). Determination of informants' traditional medicinal knowledge was done using Mann-Whitney *U* and Kruskal-Wallis tests. A total of 49 medicinal plants used in treating female reproductive health-related syndromes across four categories were recorded. Significant differences in traditional medicinal knowledge were recorded when informants were grouped according to age, education, and number of children. Issues discussed in this research could hopefully raise awareness on changes in healthcare practices in indigenous cultures and on medical safety especially when traditional and conventional medications interact.

## 1. Introduction

Cultural traditions of healthcare among women during pregnancy, birth, postpartum, and neonatal periods are common in Southeast Asia. These traditions in many rural areas in this region form the core of women's primary healthcare [1] because women are the most frequent users of complementary and alternative medicine (CAM) and herbal preparations [2]. In particular, traditional herbal therapies for women's reproductive health are numerous in this developing region and have been recorded in previous ethnographic studies [3–5].

In the Philippines, one of the many ethnic minorities with rich traditional knowledge about the use of medicinal plants and herbal medications are the Ati Negrito people of Guimaras Island [6]. The Negritos (Spanish for “little black people”), wherein the Ati is a subgroup, were the first to inhabit the Philippine archipelago prior to the arrival of other ethnic tribes from mainland Asia and of the Spanish colonizers. Even today, the Ati Negrito people (Ati hereafter) still subsist on the hunting and gathering of forest products which are at times supplemented by occasional subsistence farming and wage labor [7]. The ethnic group from central Philippines however is more particularly recognized for having good knowledge of traditional medicine (TM) and as peddlers (ambulant vendors) and traders of* materia medica* [8, 9]. This implies that Ati traditional medications, even though classified as noncodified, are being practiced not only by the women within the group, but also by non-Ati locals who patronize Ati crude herbal products. According to the Beijing Declaration (during the WHO Congress on Traditional Medicine), TM, when adopted by other populations outside its indigenous or traditional culture, is often called CAM [10].

However, the use of CAM, although it is common among women of reproductive age, still has unresolved underlying mechanisms behind its effects, if not lacking strong evidence of effectiveness [11]. Further, the wide use of traditional remedies, especially from noncodified pharmacopoeia, leaves many questions unanswered (e.g., safety concerns, relation to major health issues, and sustainability of supply) [12]. A shift in knowledge from traditional toward a pluralistic medical system that incorporates both traditional and conventional medicine is also promoted with the introduction of biomedicine. In a more unfortunate case, it may involve the loss of traditional knowledge and even its practitioners [13].

Various factors may have spurred the changes in women's traditional healthcare practices in the Philippines. The main driver for this cultural transition is maternal mortality. Studies in the past decades reported high maternal mortality especially for women in rural areas, indicating poor reproductive health services and suggesting the need for more efficient intervention strategies to reach the underserved subgroups [14, 15]. Through the UN Millennium Development Goals 5 (MDGs5), the country began to strengthen its commitments in reducing maternal mortality ratio which since then dropped from 170 (per 100,000 live births) in 1990 to 99 deaths in 2010 [16]. In early 2014, a year before the MDGs5 target deadline, the country approved its first ever reproductive health (RH) bill giving a way to improve women's healthcare by introducing modern methods of family planning and medical care. Similarly, social welfare projects like the conditional cash transfer (CCT) program encourage the formation of self-help attitude among poor families to improve their own health, education, and economic conditions in return for financial aid. Beneficiaries of the program (parents and pregnant women as representatives) are required to participate in the development sessions especially regarding health and family planning [17].

The conditions of having low socioeconomic status highly qualify ethnic minorities, such as the Ati women of Guimaras Island to these modern healthcare and social welfare programs, which we think are challenging and gradually transforming ethnomedicinal traditions. These factors motivated us to conduct this research with the specific aims (1) to document the herbal therapies for female reproductive healthcare and their cultural importance; (2) to evaluate the informants' traditional medicinal knowledge in this aspect; and (3) to raise issues about the implications of social-health policies on indigenous women's health and traditional healthcare practices.

## 2. Materials and Methods

### 2.1. Study Site and Informants

**Figure 1:**
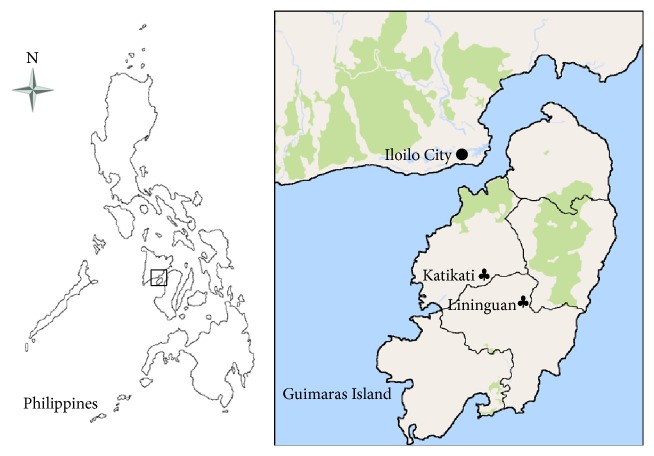
The study sites (*♣*) and the neighboring area for plant trade (●).

The study was conducted in two Ati communities of about 80 households. The communities are officially recognized as indigenous by the National Commission for Indigenous Peoples (NCIP) Region 6-7, the agency which protects the welfare of indigenous and ethnic minorities in central Philippines, including those in the study area, Guimaras Island. The island province lies between 10°25′00′′ and 10°46′09′′ north latitude, and 122°28′20.99′′ and 122°28′40.53′′ east longitude, with a great part of its land area about 100 meters above the mean sea level. The doctor to population ratio on the island is over than the standard of 1 : 20,000, while the rural health midwife ratio of 1 : 2,520 is lower than the standard of 1 : 5,000, indicating that there are more than enough midwives to handle deliveries and birth [18]. Both communities are located near (within 2 kilometers) elementary and high schools, and hospital or health centers. The research area is commercially close to the capital of the region, Iloilo City, where the majority of Ati herbal medicines are sold by Ati vendors and traders.  Figure 1 shows the map of the study sites and the neighboring area for plant trade.

A total of 36 Ati women, each one representing a single household aged 18 to 80 years, were selected as informants. Samples were grouped according to age, educational level, and number of children for statistical comparability and computation. Approximately 67% of the women are subscribed to the national insurance service (or covered by the short-term local insurance program), while about 56% are beneficiaries of government financial assistance. The data presented here were drawn from the first author's master's thesis which was conducted from 2013 to 2014 [6]. The research followed legal procedures set by the NCIP and regulations by the tribe council following mutually agreed terms.

Only informants who accepted the request for interview became part of the study and were asked using semistructured questionnaires in a local language, which the Ati people more commonly use, and the primary author's mother tongue. Interviews were conducted separately to minimize the possibility of one informant's answer directly influencing another's. Only the informants' personal experiences in the direct application or assistance in herbal preparations were recorded. It was unfortunate however that none of the informants was a traditional midwife (“hilot”) since the last practitioner had died a few years before the research was conducted. Nevertheless, we were able to interview eleven key informants (*n* = 11) who were either medicinal plant gatherers or were involved in the preparation and selling of herbal medicine. The remaining twenty-five participants represented different sociocultural roles and occupations such as weavers, housekeepers, and students (*n* = 25).  Table 1 presents the informants' demographic data.

Plant specimens were collected together with the key informants or when possible with the nonexpert participants themselves for identification purposes. Pictures (and videos) on how some plants are prepared into crude herbal products and on how some therapies are administered were also taken when given consent. Local names of plants and indigenous terms of their uses were also documented by the first author during the data collection period which required direct community observation and participation. Samples of plants were pressed, dried, and brought to South Korea for taxonomic documentation in compliance with phytosanitary requirements. After plant scientific names were determined, specimens were deposited as vouchers at the Herbarium of Hallym University (HHU).

### 2.2. Quantitative Data Analyses

To evaluate and compare informants' knowledge about medicinal plants and phytotherapies, use-reports were computed and analyzed using PASW Statistics 18 software [19]. Nonparametric inferential statistics Mann-Whitney *U* and Kruskal-Wallis tests were used to determine significant difference(s) involving two and three related groups, respectively. All statistics were set at 0.05 level significance.

To determine the relative cultural importance of plants and herbal therapies, Fidelity Level (FL) was utilized. FL is a quantitative ethnobotanical index based on informant consensus method. This index assumes that citation frequency is an indicator of importance and effectiveness of phytotherapies. It is the ratio between the number of informants who suggested the use of a plant for a particular purpose (herein termed as use-mention) and the total number of informants who mentioned the use of plant for any purpose [20]. It is calculated using the following formula: FL (%) = (*I*_*p*_/*I*_*u*_) × 100, where *I*_*p*_ is the number of informants who independently suggested the use of a plant for a particular purpose and *I*_*u*_ is the total number of informants who mentioned the plant for any purpose.

### 2.3. Reproductive Health-Related Categories

To facilitate the computation of relative cultural importance of medicinal plants and phytotherapies, we established four categories based on the reported reproductive health-related syndromes found below.

#### 2.3.1. Menstruation-Related Category

Plant therapies reported in this category are used to treat (a) dysmenorrhea and (b) delayed menstruation syndromes. Dysmenorrhea is a condition characterized by pain during menstruation, while delayed menstruation syndromes, such as amenorrhea, are disorders associated with changes in the length of menstrual cycle [21]. In this category, some symptoms of delayed menstruation (e.g., abdominal pains) may overlap with those of dysmenorrhea.

#### 2.3.2. Birth/Delivery-Related Category

Plants and therapies used in this category are employed as (a) delivery inducers or as (b) tools during birth. Health conditions similar to the latter are categorized by the WHO International Classification of Diseases 10 as factors influencing health status and contact with health services [22]. People with low socioeconomic conditions are believed to rely more on traditional therapies because of inaccessibility to healthcare services [23].

#### 2.3.3. Postpartum-Related Category

Phytotherapies in this category are employed to stop bleeding after childbirth and cleanse the womb from unwanted blood and impurities, among other perceived symptoms. The subcategories are postpartum-related (a) abdominal pain; (b) headache; (c) hemorrhage; (d) postpartum relapse, a setback that occurs during period of health progress; and (e) postpartum wash applications.

#### 2.3.4. Neonatal Care-Related Category

Plant therapies in this category are applied as (a) galactagogues, substances that increase the production or flow of milk and (b) newborn baby care applications. Some herbal therapies for neonatal care include remedies to expulse infant's swallowed discharges during delivery and other perceived illnesses, or as infant wash preparations. Therapies administered during growth from infanthood to babyhood are not discussed here.

## 3. Results and Discussion

### 3.1. Reproductive Health Herbal Therapies

In this part, we present the plants and phytotherapies which recorded the highest consensus from informants (by counting use-mentions), discuss their cultural importance, and present related studies supporting (or refuting) their claimed effectiveness or safety in treating reproductive health-related syndromes. Implications of social and healthcare policies on informants' traditional healthcare practices are also discussed.

#### 3.1.1. Plants and Phytotherapies for Menstruation-Related Syndromes

A total of 7 plant species for treating (a) dysmenorrhea and (b) delayed menstruation syndromes were reported in this category. The plants which recorded the highest informant consensus are discussed as follows.

(a)* Catharanthus roseus*, administered as leaf decoction in treating dysmenorrhea, showed the highest informant consensus. The analgesic properties of* C. roseus* are probably due to its alkaloids and chemotherapeutic agents which are also known for their anticancer pain-relieving properties [24]. According to Oats and Abraham [21], plants with analgesic effects can provide temporary relief from dysmenorrhea, as well as other aches like abdominal, back, pelvic, and even severe labor pains.

(b)* Lunasia amara* bark, infused in local rum and orally administered to treat delayed menstruation syndromes, including abdominal pain, a common reported symptom, recorded the highest use-mention. According to Bowman et al. [25],* Lunasia* species contain quinoline alkaloids which possess pain-relieving qualities. Quinoline alkaloids (from a* Lunasia* related genus) have shown to possess characteristics of estrogenic activity (e.g., causing the uterus to hydrate, changing menstrual cycle) in an animal study in mature intact rat [26]. Animal and clinical studies are therefore necessary to confirm if similar properties of these compounds found in* L. amara* also show estrogenic properties.

It is highly probable however that plants used for menstruation-related syndromes possess muscle-relaxing characteristics. These plants were found to be uterine spasmolytics which alleviate uterine cramps and uterine spasmogenics which ease menstrual pains by inducing the menses [27]. Plants that disrupt the estrous cycle have also shown contraceptive effects, and many plants that are used for the treatment of amenorrhea or those that function as emmenagogues are more likely taken as early-stage abortifacients [1]. Harlow and Campbell [28] reported that there are indications that emmenagogues are commonly used to treat dysmenorrhea in low-income countries where proper facilities to determine pregnancy are most often lacking.

There is high possibility that most reported plants in this category can cause abortion since all applications are taken orally, some in pure concentration, others infused in liquors with high alcohol content. The use of these potential herbal abortifacients should be carefully considered because most often this leads to serious consequences for women. About 13% of maternal deaths are attributed to unsafe abortions in Southeast Asia [29], and hospitalization of women due to induced abortion in the Philippines was estimated at 473,400 in 2000, with numbers for the central region (where Ati* materia medica* is sold) showing an increase by 63% [30].

During our interview, participants willingly disclosed the plants used in treating menstruation-related syndromes, but they became hesitant to answer when asked if the same plants were also used as contraceptives or abortifacients because assisting or participating in abortion is illegal (nonetheless practiced) in predominantly Catholic Philippines. Some admitted that plants and oral remedies with bitter taste are taken as contraceptives but could trigger abortion when taken in high dosage. The same informants however clarified that the therapy is not being practiced by Christianized Ati women.

The implementation of the RH bill is also seen to influence how Ati women and couples plan the number of children they desire as education on contraception options and contraceptives like pills are provided in government health centers free of charge. These provisions however are seen to have negative implications on the use of TM and related herbal therapies, not to mention the dangers when uninformed practitioners combine conventional medications with traditional herbal treatments. According to Lai et al. [31], many women do not disclose their complementary therapies to their physicians, and since treatment plans are often not coordinated, the risk of adverse events during interactions of complementary with conventional therapies is high.

#### 3.1.2. Plants and Phytotherapies for Childbirth/Delivery

Only 3 medicinal plants used as (a) delivery inducers or as (b) tools during birth were reported in this category and are all discussed below.

(a)* Mucuna pruriens*, believed to hasten delivery, is administered by applying its scraped bark and stem onto the patient's abdomen. Related studies reported that cultures from* M. pruriens* have been shown to accumulate high levels of L-DOPA, a precursor substance to catecholamines like dopamine, norepinephrine, and epinephrine (adrenaline) [32]. According to Odent [33], increased levels of catecholamines activate the fetal ejection response during childbirth.

*Corchorus olitorius* is similarly believed to hasten childbirth when slippery components of its crushed leaves are rubbed on the gestating patient who is about to give birth. In Nigeria, however, the leaves of* C. olitorius* are taken orally to treat delayed and prolonged labor [34]. This implies that properties in the leaves may have effects on muscle contraction and relaxation, as clinical experiments using its sister species,* C. depressus*, showed direct relaxing effect on rabbit smooth muscle [35].

The slimy properties of parts of* M. pruriens* and* C. olitorius* used to speed up labor are without doubt the reason why participants have preferred these plants as remedies. Unlike other* partus preparators* which are administered orally and taken from a few days to a month before the suspected due date [36], applications of* M. pruriens* and* C. olitorius* are externally administered and applied only at the time of labor. This leaves questions on whether agent compounds enter the bloodstream and facilitate delivery. The attribution of their therapeutic powers is more probably in accordance with the ideas found in the Doctrine of Signatures, a theory in old natural philosophy and not science. Further trials should therefore be conducted to determine whether chemical constituents contain properties that augment labor during delivery.

(b)* Schizostachyum lumampao* is the only plant used as an instrument in cutting the umbilical cord that connects the mother and infant. Accordingly, it was commonly used as a tool by traditional birth attendants during home birthing services especially in the past when government health facilities were not accessible to Ati communities. The use of this plant as tool is similarly practiced by other indigenous groups in the Philippines [37].

These days, however, young Ati women and mothers prefer the hospital as a place to give birth for convenience and safety reasons. If residence is rather remote during labor, the patient has the option to ask for services of a medically trained midwife (“paltera”) who is most often non-Ati. Pregnant women who are beneficiaries of the government financial aid program however are strictly required to give birth in hospitals or birthing centers and to undergo prenatal and postnatal checkups [38]. Although this policy was implemented to lessen maternal mortality during childbirth, the same regulation may also be gradually undermining the profession of traditional birth attendants in ethnic communities. Efforts to integrate traditional health workers to formal health service force, or at least encourage their cooperation, should therefore be discussed.

#### 3.1.3. Plants and Phytotherapies for Postpartum-Related Syndromes

A total of 31 medicinal plants for the treatment of postpartum-related syndromes such as (a) abdominal pain, (b) headaches, (c) hemorrhage, (d) postpartum relapse, or (e) postpartum wash applications were reported in this category. The plants which recorded the highest informant preference are discussed below.

(a)* Blumea balsamifera* leaves, externally administered as hot compress, recorded the highest number of use-mention in treating postpartum abdominal pain.* B. balsamifera* has also been recorded as one of the main ingredients in hotbed/steam bath for postpartum recovery therapy in Laos [39]. Adding* B. balsamifera* to bath could have conferred some antibacterial and antifungal effects due to the presence of these properties in the essential oils and extracts of this plant [40].

(b)* Canarium asperum*, on the other hand, recorded the highest use-mention in treating postpartum-related headaches when its bark resin is administered either in aromatherapy or taken as decoction. Members of genus* Canarium* are known for the use of their essential oil-containing resin [41], which is perhaps the factor why the plant is similarly used as cure for headaches by other ethnic groups in the Philippines [42].

In applications like steam baths, hot compress, or aroma therapies, essential oils can be absorbed through the skin or by aromatic inhalation, where they travel through the bloodstream, stimulate brain functions, and promote whole-body healing [43]. Potential safety concerns however may arise when oil extracts from plants are not well diluted, consequently causing skin irritations. Reports have supported the negative interaction with conventional medicine of these oils, both ingested and applied to the skin [44].

(c)* Ixora philippinensis* and* Ardisia elliptica* equally recorded the same number of use-mentions and are both taken as decoction in treating postpartum hemorrhage (PPH). Genus Ixora members have shown to have antiulcer and anti-inflammatory activity and are also used for hemorrhage treatments in Indian TM [45, 46].* A. elliptica* components, on the contrary, were observed to have inhibiting effects on platelet aggregation [47]. Although informants could not further elaborate the relation of both plants in treating PPH, the seemingly contrasting effects of the two imply their balancing/counteracting effects as both are usually administered at the same time but in separate preparations. The use of these plants in treating postpartum bleeding must therefore be carefully examined since PPH is still the most common cause of maternal death that accounts for 35% in developing regions [48] and nearly 25% worldwide [49].

(d)* Uvaria grandiflora* recorded the highest use-mention when its chopped stems are boiled and administered as hot compress in treating postpartum relapse (“bughat”). The medical condition (or perhaps a Filipino medicocultural concept) is described by locals as a sudden feeling of sickness occurring after perceived recovery. It is usually triggered when the patient exerts force or is exposed to cold elements while still recuperating. This condition may include general symptoms (e.g., fever, headache, and body pains) observed when the patient is sick or is recuperating during early period of recovery. Assays of* U. grandiflora* showed that flavonoids may be responsible for the strong antibacterial activity of ethanol extracts found in its bark [50].

(e)* Bambusa vulgaris* on the other hand recorded the highest use-mention when its leaves are boiled and applied as wash or hot compress. Its common distribution and availability throughout the year and the antimicrobial activity of its leaves [51] may have been the factor for its high preference. Ati postpartum wash phytotherapy is prepared by boiling 7 pieces of a particular plant part (usually the leaves) taken from 7 different plant species (see Table 2). The number is a significant figure for Ati people when preparing general remedies and is also being practiced by other indigenous cultures in the Philippines [52].

We have observed that reported herbal therapies for postpartum relapse and preparations for postpartum wash have overlapping perceived efficacy. For example, hot compress and wash applications, drawn from different plant candidates and prepared as mixture, are also administered to prevent the occurrence of postpartum relapse. The substitution of a particular plant for another may imply that the replaced plant has little (or no) therapeutic properties and may have been selected only due to its accessibility in the first place. These signs of merely having placebo-like effects of therapies concerning women's reproductive health are occurring even in codified TM [53, 54] and may not far be the case of some applications in this investigation. What is clear however is that some phytotherapies presented here are complex and have attached cultural and spiritual meanings only the participants could understand. According to Moerman and Jonas [55], botanical medicinal effectiveness across cultures is some varying combination of pharmacology and symbolic meaning.

In comparison with other Southeast Asian postpartum therapies [4, 56], those by the Ati seem less elaborate. Recuperation after childbirth only takes 9 days and perhaps shorter among the past generations. This relatively shortened period of recovery is seen as a characteristic of the once nomadic people who would constantly move from place to place to hunt and forage. Elderly informants during our interview, however, expressed concerns about the gradual transition of traditional postpartum practices to modern healthcare medication because young mothers these days consider the therapies troublesome and lengthy. Government regulations have also been encouraging all pregnant women, regardless of the ethnicity, to give birth in hospitals or at least in birthing centers [57]. These policies seem to have led indigenous women to a dilemma, a difficult situation in which a choice between conventional or traditional medications is made.

#### 3.1.4. Plants and Phytotherapies for Neonatal Care

A total of 11 plants used as (a) galactagogues or used for (b) newborn baby care were reported in this category. The plants that recorded the highest consensus from informants are discussed below.

(a)* Ficus nota *stem decoction, administered orally as milk production enhancer, recorded the highest use-mention. A recent review of traditional remedies for women's healthcare in Southeast Asia has also documented the preference for* Ficus* species as galactagogues [1]. Systematic reviews of published studies however found lack of evidence for herbal galactagogue effectiveness, including those found in pharmaceutical literatures [58, 59]. Informant consensus in selecting* F. nota* as galactagogue appears to be due to the milky white latex found in its stems (and many of its organs). The selection of the plant and its application seem to be based on the Doctrine of Signatures, which has recently been described only as post hoc attributions and mnemonics [60].

(b)* Physalis angulata* recorded the highest use-mention when its heated leaves are applied on infant stomach as poultice to treat the neonate's greenish diarrhea (“balaud”), technically termed meconium. Meconium is the newborn dark green viscous first stool, a collection of debris consisting of desquamated cells, amniotic fluid, and various intestinal secretions [61]. Meconium passage in newborns is a normal programmed event after birth indicating that the use of* P. angulata* may not exactly have antidiarrheal qualities [62]. However, its antinociceptive and anti-inflammatory properties which reduce sensitivity to painful stimuli [63, 64] may have the analgesic-like effect to the neonate suffering from digestive problems. Nevertheless, the use of any plant extract as neonatal emetic should be carefully considered, better yet discouraged, as in the application of* Momordica charantia* to remove a newborn's swallowed lochia. Potential fatal reaction leading to hypoglycemic coma after ingestion of its leaf and stem extracts were reported even in 3- and 4-year old children [65].

As an effort to lessen neonatal death in the Philippines, children (up to 5 years old) from families that are beneficiaries of the conditional cash transfer program are required to undergo regular health checkups and to get vaccinated. In return, the families receive financial assistance (about 11 to 32 USD) for health, nutrition, and education per month, depending on the number of eligible children per household [17]. This strategy is seen to significantly lower neonatal, infant, and under-five mortality rates in the Philippines, which was 14, 24, and 29 deaths per 1,000 live births, respectively, as those of 2013 [66].

### 3.2. Characteristics of Plants and Herbal Preparations

Over all, this study was able to identify 49 plant taxa used in 4 categories concerning Ati women's reproductive health syndromes. The most frequently used plant parts were the leaves (49%), stems (38%), and barks (6%) perhaps due to the availability of these aerial organs all year round in tropical Philippines. Botanically, most leaves, stems, and barks contain phytochemicals which act as toxins protecting the plant from herbivores, but we humans economically utilize them as medicines. External administration (52%) was slightly preferred to internal one (48%) more likely due to safety concerns and ease of preparation.

A total of 37 plant taxa recorded 100% FL values indicating the importance and therapeutic effectiveness of these plants. The species which recorded the highest use-mentions were* Canarium asperum* (24),* Bambusa vulgaris* (24),* Gliricidia sepium* (22),* Physalis angulata* (22), and* Blumea balsamifera* (21). FL for plants reported by only a single participant was not computed due to the lack of consensus. High FL values are obtained for plants for which almost all use-mentions refer to the same purpose; that is, the plants (and their use in therapies) were most preferred implying the effectiveness of herbal remedy. Table 2 presents the taxonomic information, preparation and administration, and relative cultural value of all reported plants.

### 3.3. Traditional Medicinal Knowledge

When grouped according to education, descriptive and inferential statistics revealed that informants with lower level (none to complete elementary) of education (*Md* = 14, *n* = 18) recorded higher concordance in the use of herbal therapies than informants with higher level (secondary to tertiary) of education (*Md* = 7.50, *n* = 18), as shown in Mann-Whitney *U* test (*U* = 77, *p* < 0.01). The findings imply that the latter group is more likely exposed to conventional medicine and information about bioscience as these are formally taught in schools. A rather more agreeable explanation is that all the interviewed key informants (herbalists and experts in Ati TM) belong to the group with lower educational level.

When grouped according to age, results revealed that informants from the age group of 49 years and above (*Md* = 16.50, *n* = 12) showed the highest concordance in the use of phytotherapies as compared to informants from the age groups of 30 to 48 years (*Md* = 11, *n* = 12) and 18 to 29 years (*Md* = 2, *n* = 12), as shown in Kruskal-Wallis test (*x* ^2^ (2, *n* = 36) = 23.99; *p* < 0.001). The findings, as we expected, were due to the degree of experience as informants increase in age, in addition to varying generational, social, and cultural experiences which have most probably influenced not only traditional medicinal knowledge but also attitudes.

When grouped according to the number of children, statistics revealed that informants with 6 and more children (*Md* = 18.50, *n* = 10) recorded the highest concordance in herbal applications as compared to groups with 3 to 5 (*Md* = 11, *n* = 11) and none to 2 children (*Md* = 2, *n* = 15), as shown in Kruskal-Wallis test (*x* ^2^ (2,  *n* = 36) = 17.87; *p* < 0.001). Women with more children have had more direct experience in giving birth and therefore have more practical knowledge in related medications. As in most cultures, Ati mothers are also the ones expected to take care of sick children or assist other women who are about to give birth. It is also worth mentioning that the number of children is directly related to the age of informants, and either age or number of children could explain the significant differences in traditional knowledge when grouped accordingly.

Statistical limitations of the analyses discussed above, however, are acknowledged by the authors. First, since the interview of indigenous people was bound by free and prior informed consent ethics, random sampling could not be applied. Second, the inferences made on informants' knowledge in TM do not attempt to decontextualize their deeper understanding of culturally established phytotherapies. The interpretations, however, may aid concerned organizations in creating programs to protect ethnomedicinal traditions.

## 4. Conclusions

This research not only presents the diversity of medicinal plants used by the Ati women in traditional herbal medicine, but also emphasizes the cultural importance of plants and phytotherapies used for women's reproductive health. Review of related studies on medicinal plants which recorded the highest informant consensus was also carried out to provide additional information regarding their botanical efficacy, safety, and mechanism of action when available. We hope that the study could stimulate social and cultural interests about the implications of changes happening in indigenous peoples' traditional healthcare practices and, more importantly, raise awareness on safety concerns when TM is applied together with conventional medicine. Nevertheless, we believe that the need for improvement on health services for the safety of women during menstruation, pregnancy, delivery, and postpartum periods should be the first priority especially to the seemingly underserved indigenous cultures in the country.

## Figures and Tables

**Table 1:** Demographic data of the informants.

Information	*n* (%)
Education	
None to complete elementary	18 (50)
Secondary to tertiary	18 (50)
Age	
18 to 29	12 (33.33)
30 to 48	12 (33.33)
49 and above	12 (33.33)
Number of children	
0 to 2	15 (41.67)
3 to 5	11 (30.56)
6 and above	10 (27.78)

**Table 2:** Ati Negrito medicinal plants and phytotherapies for female reproductive healthcare.

Reproductive health category	Plant scientific (and family) name	Local name	Preparation and administration	Use-mention	FL (%)
Menstruation syndromes					
(a) Dysmenorrhea	*Catharanthus roseus *(L.) G.Don (Apocynaceae)	Rosas de baybayon	Oral application after decocting leaf	19	100
	*Swietenia mahogani *L. (Meliaceae)	Mahogani	Oral application after powdering dried seed	11	100
	*Lunasia amara *Blanco (Rutaceae)	Kamias	Oral application as tonic after infusion of bark in local rum	10	50
	*Alstonia scholaris* (L.) R. Br. (Apocynaceae)	Bita	Oral application after powdering dried bark	9	100
	*Arcangelisia flava *(L.) Merr. (Menispermaceae)	Albutra	Oral application as tonic after infusion of dried stem in alcohol	6	37.5
	*Lantana camara *L. (Verbenaceae)	Haroy-haroy	Oral application after decocting leaf	5	38.46
					
(b) Delayed menstruation	*Lunasia amara *Blanco (Rutaceae)	Kamias	Oral application after infusion of bark with *A. flava* in alcohol	10	50
	*Arcangelisia flava *(L.) Merr. (Menispermaceae)	Albutra	Oral application after infusion of stem with *L. amara* in alcohol	10	62.5
	*Lantana camara *L. (Verbenaceae)	Haroy-haroy	Oral application after decocting leaf	5	38.46
	*Tinospora crispa* (L.) Hook.f. & Thomson (Menispermaceae)	Manunggal	Oral application of fresh stem extract	5	100

Birth/delivery uses					
(a) Delivery inducer	*Mucuna pruriens *(L.) DC. (Fabaceae)	Nipay	Topical application of scraped bark and stem mixed with coconut oil	3	100
	*Corchorus olitorius *L. (Malvaceae)	Tugabang	Topical application of crushed leaves	2	100
					
(b) Delivery tool	*Schizostachyum lumampao *(Blanco) Merr. (Poaceae)	Bagakay	Stem used as tool in cutting umbilical cord	17	73.91

Postpartum syndromes					
(a) Abdominal pain	*Blumea balsamifera* (L.) DC (Asteraceae)	Alibhon	External application as wash or hot compress after boiling leaves	21	53.85
	*Salacia *sp. (Celastraceae)	Montawi	Oral application after decocting dried stem	11	100
	*Rauvolfia amsoniifolia* A.DC. (Apocynaceae)	Agoparit/Magoparit	Oral application after decocting dried stem	7	100
	*Chrysophyllum cainito *L. (Sapotaceae)	Star apol	External application as wash or hot compress after boiling leaves	6	50
			Oral application after decocting leaves	6	50
	*Tabernaemontana pandacaqui *Lam. (Apocynaceae)	Alibotbot	Topical application of heated leaves on abdomen as poultice	6	100
	*Cajanus cajan *(L.) Millsp. (Fabaceae)	Kadios	Topical application of crushed leaves as poultice	3	100
(b) Headache	*Canarium asperum *Benth. (Burseraceae)	Salong	Oral application after decocting dried stem resin	24	50
			External application as aromatherapy by burning dried stem resin	24	50
	*Justicia gendarussa *Burm. F. (Acanthaceae)	Bunlaw	External application as wash or hot compress after boiling leaves with *S. elliptica *	15	100
	*Schefflera elliptica *(Blume) Harms (Araliaceae)	Kamoy-kamoy/Kalangkang	External application as wash or hot compress after boiling leaves and stems with *J. gendarussa *	10	100
	*Blumea balsamifera* (L.) DC (Asteraceae)	Alibhon	Oral application after decocting leaves with *F. pseudopalma *	7	17.95
	*Ficus pseudopalma *Blanco (Moraceae)	Sulamyog	Oral application after decocting stems with *B. balsamifera *	7	50
	*Ficus nota* (Blanco) Merr.	Tabuyog	Oral application after decocting dried stems	6	33.33
	(Moraceae)				
	*Vitex trifolia *subsp. *litoralis* Steenis (Lamiaceae)	Lagundi	Oral application after decocting leaves	4	100
					
(c) Hemorrhage	*Ardisia elliptica *Thunb. (Primulaceae)	Tagpo-bayi	Oral application after decocting dried stems	8	100
	*Ixora philippinensis *Merr. (Rubiaceae)	Tagpo-laki	Oral application after decocting dried stems	8	100
	*Caesalpinia sappan *L. (Fabaceae)	Sibukaw	Oral application after decocting dried stems	3	100
					
(d) Postpartum relapse	*Uvaria grandiflora* Roxb. (Annonaceae)	Saging-saging/Kalansaging	External application as wash or hot compress after boiling stems	16	100
	*Cymbopogon schoenanthus *(L.) Spreng. (Poaceae)	Tanglad	External application as wash or hot compress after boiling whole plant	11	100
	*Uvaria rufa *Blume (Annonaceae)	Banawak	Oral application after decocting dried stems	5	26.32
	*Premna odorata *Blanco (Lamiaceae)	Adgaw/Agdaw	External application as hot compress after boiling leaves	4	100
	*Corypha utan *Lam. (Arecaceae)	Buri	External application as hot compress after boiling young shoots	3	100
	*Embelia whitfordii *Merr. (Primulaceae)	Malaumau	Oral application after decocting dried stems and leaves	3	100
	*Smilax bracteata *C.Presl (Smilacaceae)	Banagan	Oral application after decocting stems	1	—
(e) Postpartum wash	*Bambusa vulgaris *Schrad.^*∗*^ (Poaceae)	Kawayan	External application as wash or hot compress after boiling leaves	24	100
	*Gliricidia sepium* (Jacq.) Walp. (Fabaceae)	Madre cacao	External application by sitting on heated leaves to remove discharges	22	100
	*Citrus maxima* (Burm.) Osbeck^*∗*^ (Rutaceae)	Kabugao	External application as wash or hot compress after boiling leaves	19	100
	*Uvaria rufa *Blume^*∗*^ (Annonaceae)	Banawak	External application as wash or hot compress after boiling dried stems	14	73.68
	*Pittosporum pentandrum* (Blanco) Merr.^*∗*^ (Pittosporaceae)	Balingkawayan	External application as wash or hot compress after boiling leaves	12	100
	*Ficus pseudopalma *Blanco^*∗*^ (Moraceae)	Sulamyog	External application as wash or hot compress after boiling stems	7	50
	*Antidesma bunius *(L.) Sreng.^*∗*^ (Phyllanthaceae)	Bugnay	External application as wash or hot compress after boiling leaves	6	100
	*Leucaena leucocephala* (Lam.) de Wit^*∗*^ (Fabaceae)	Agho	External application as wash or hot compress after boiling leaves	4	100
	*Areca catechu* L. (Arecaceae)	Bunga	External application as wash or hot compress after boiling leaves	3	100
	*Lantana camara *L.^*∗*^ (Verbenaceae)	Haroy-haroy	External application as wash or hot compress after boiling leaves	3	23.08
	*Morinda citrifolia *L.^*∗*^ (Rubiaceae)	Anino	External application as wash or hot compress after boiling dried stems	3	100

Neonatal care uses					
(a) Galactagogue	*Ficus nota* (Blanco) Merr. (Moraceae)	Tabuyog	Oral application after decocting stems	12	66.67
	*Moringa oleifera *L. (Moringaceae)	Balunggay	Consumed after boiling leaves (sometimes with young *C. papaya* fruit)	8	100
	*Musa balbisiana *Colla (Musaceae)	Saging (sab-a)	Topical application of young leaves on breast as poultice	6	100
	*Carica papaya *L. (Caricaceae)	Kapayas	Consumed after boiling young fruit (sometimes with *M. oleifera* leaves)	4	100
	*Ipomoea batatas *(L.) Poir. (Convolvulaceae)	Kamote (pula)	Consumed after steaming young leaves	3	100
	*Manihot esculenta*Crantz (Euphorbiaceae)	Balinghoy	Topical application of young leaves on breast as poultice	3	100
(b) Newborn baby care	*Physalis angulata* L. (Solanaceae)	Tino-tino	Topical application of heated leaves on infant stomach as poultice	22	100
	*Citrus* × *microcarpa *Bunge (Rutaceae)	Suha	External application as infant wash after boiling leaves	10	100
	*Momordica charantia *L. (Cucurbitaceae)	Margoso	Internal application of leaf extract to expulse swallowed lochia	8	100
	*Schizostachyum lumampao *(Blanco) Merr. (Poaceae)	Bagakay	Topical application on infant's freshly-cut navel of ash from burnt stem	6	26.09
	*Pandanus tectorius *Parkinson ex Du Roi (Pandanaceae)	Pandan	Topical application on infant's freshly-cut navel of ash from burnt dried leaves	1	—

^*∗*^Prepared in combination with 6 other plants.

## References

[1] De Boer H. J., Cotingting C. (2014). Medicinal plants for women's healthcare in southeast Asia: a meta-analysis of their traditional use, chemical constituents, and pharmacology. *Journal of Ethnopharmacology*.

[2] Murphy P. A., Kronenberg F., Wade C. (1999). Complementary and alternative medicine in women's health: developing a research agenda. *Journal of Nurse-Midwifery*.

[3] Chithtalath S.-A., Earth B. (2001). From the forest to the clinic: changing birth practice among the Katang, Lao. *Reproductive Health Matters*.

[4] Liulan W., Nanakorn W., Fukui K. (2003). Food and medicinal plants used for childbirth among Yunnanese Chinese in Northern Thailand. *Journal of Ethnobiology*.

[5] de Boer H., Lamxay V. (2009). Plants used during pregnancy, childbirth and postpartum healthcare in lao PDR: a comparative study of the Brou, Saek and Kry ethnic groups. *Journal of Ethnobiology and Ethnomedicine*.

[6] Ong H. G. (2014). *Ethnobotany of the medicinal plants used by the Ati Negrito indigenous group in Guimaras Island, Philippines: a quantitative approach [M.S. thesis]*.

[7] Stewart T. (1992). Land-use options to encourage forest conservation on a tribal reservation in the Philippines. *Agroforestry Systems*.

[8] Zayas C. N., Paz C. J. (2008). Trade and patronage of Ati materia medica in the Visayas. *Ginhawa, Kapalaran, Dalamhati: Essays on Well Being, Opportunity/Destiny, and Anguish*.

[9] De la Peña L. (2009). The power to influence and to protect: interconnectedness of the human bodies. *Liceo Journal of Higher Education Research*.

[10] WHO (2008). *Beijing Declaration*.

[11] Wu X., Ng E. H. Y., Stener-Victorin E., Legro R. S. (2014). Effects and mechanisms of complementary and alternative medicine during the reproductive process. *Evidence-Based Complementary and Alternative Medicine*.

[12] Bussmann R. W., Applequist W., Paniagua-Zambrana N. (2014). Traditional medicine in a global environment. *Evidence-Based Complementary and Alternative Medicine*.

[13] Smith-Oka V. (2008). Plants used for reproductive health by Nahua women in northern Veracruz, Mexico. *Economic Botany*.

[14] Acuin C. S., Javellana J., Balis A. C. (1994). The role of traditional health-care practitioners in the delivery of health care—a secondary analysis of NDS-SMS 1993 data. *Philippine Population Journal*.

[15] Westley S. B., Kantner A. (1996). Who uses reproductive health services in the Philippines (and who doesn't)?. *Asia-Pacific Population and Policy*.

[16] Yamashita T., Suplido S. A., Ladines-Llave C. (2014). A cross-sectional analytic study of postpartum health care service utilization in the Philippines. *PLoS ONE*.

[17] Fernandez L., Olfindo R. (2011). Overview of the Philippines' conditional cash transfer program: the Pantawid Pamilyang Pilipino Program (Pantawid Pamilya). *Philippine Social Protection Note*.

[18] Province of Guimaras (2008). *Province of Guimaras Provincial Development and Physical Framework Plan 2008–2013*.

[19] SPSS (2009). *PASW Statistics for Windows Version 18.0*.

[20] Friedman J., Yaniv Z., Dafni A., Palewitch D. (1986). A preliminary classification of the healing potential of medicinal plants, based on a rational analysis of an ethnopharmacological field survey among Bedouins in the Negev Desert, Israel. *Journal of Ethnopharmacology*.

[21] Oats J., Abraham S. (2010). *Llewellyn-Jones Fundamentals of Obstetrics and Gynaecology*.

[22] World Health Organization (2011). *International Statistical Classification of Diseases and Related Health Problems*.

[23] Gaitonde B., Kurup P., Bodeker G., Ong C., Grundy C. (2005). Regional overview: south-east Asia region. *WHO Global Atlas of Traditional, Complementary and Alternative Medicine*.

[24] Govindasamy C., Srinivasan R. (2012). In vitro antibacterial activity and phytochemical analysis of *Catharanthus roseus* (Linn.) G. Don.. *Asian Pacific Journal of Tropical Biomedicine*.

[25] Bowman R. M., Gray G. A., Grundon M. F. (1973). Quinoline alkaloids. Part XV. Reactions of a quinoline isoprenyl epoxide with hydride reagents. Asymmetric synthesis and stereochemistry of lunacridine and related *Lunasia* alkaloids. *Journal of the Chemical Society, Perkin Transactions 1*.

[26] Nazrullaev S. S., Bessonova I. A., Akhmedkhodzhaeva K. S. (2001). Estrogenic activity as a function of chemical structure in *Haplophyllum quinoline* alkaloids. *Chemistry of Natural Compounds*.

[27] Van Andel T., De Boer H. J., Barnes J., Vandebroek I. (2014). Medicinal plants used for menstrual disorders in Latin America, the Caribbean, sub-Saharan Africa, South and Southeast Asia and their uterine properties: a review. *Journal of Ethnopharmacology*.

[28] Harlow S. D., Campbell O. M. R. (2004). Epidemiology of menstrual disorders in developing countries: a systematic review. *BJOG*.

[29] World Health Organization (2012). *Unsafe Abortion Incidence and Mortality: Global and Regional Levels in 2008 and Trends during 1990–2008*.

[30] Juarez F., Cabigon J., Singh S., Hussain R. (2005). The incidence of induced abortion in the Philippines: current level and recent trends. *International Family Planning Perspectives*.

[31] Lai J. N., Chen P. C., Wang J. D., Wu T. C., Chung V. (2015). Integrative gynecology and women’s healthcare. *Evidence-Based Complementary and Alternative Medicine*.

[32] Brain K. R. (1976). Accumulation of L-DOPA in cultures from *Mucuna pruriens*. *Plant Science Letters*.

[33] Odent M., Odent M. (1992). The fetus ejection reflex. *The Nature of Birth and Breastfeeding*.

[34] Borokini T. I., Ighere D. A., Clement M. (2013). Ethnobiological survey of traditional medicine practices in Oyo State. *Journal of Medicinal Plants*.

[35] Kataria S., Kaur D., Rao S. K., Khajuria R. K. (2013). *In vitro* and *in vivo* aphrodisiac properties of *Corchorus depressus* Linn. on rabbit corpus cavernosum smooth muscle relaxation and sexual behavior of normal male rats. *Journal of Ethnopharmacology*.

[36] Low Dog T. (2009). The use of botanicals during pregnancy and lactation. *Alternative Therapies in Health and Medicine*.

[37] Banwa T. P., Bawer M. C. C. (2013). *Schizostachyum lumampao* (byuyu): its diverse ethno-botanical uses by Lubuangan subtribe of Kalinga in North Luzon Philippines. *European Scientific Journal*.

[38] Reyes C. M., Tabuga A. D. (2012). Conditional cash transfer program in the Philippines: is it reaching the extremely poor?. *Philippine Institute for Development Studies*.

[39] Lamxay V., de Boer H. J., Björk L. (2011). Traditions and plant use during pregnancy, childbirth and postpartum recovery by the Kry ethnic group in Lao PDR. *Journal of Ethnobiology and Ethnomedicine*.

[40] Sakee U., Maneerat S., Cushnie T. P. T., De-Eknamkul W. (2011). Antimicrobial activity of *Blumea balsamifera* (Lin.) DC. extracts and essential oil. *Natural Product Research*.

[41] Mogana R., Wiart C. (2011). *Canarium* L.: a phytochemical and pharmacological review. *Journal of Pharmacy Research*.

[42] Langenberger G., Prigge V., Martin K., Belonias B., Sauerborn J. (2009). Ethnobotanical knowledge of Philippine lowland farmers and its application in agroforestry. *Agroforestry Systems*.

[43] Gaware V., Nagare R., Dhamak K. B. (2013). Aromatherapy: art or science. *International Journal of Biomedical Research*.

[44] Valnet J., Tisserand R. (1990). *The Practice of Aromatherapy: A Classic Compendium of Plant Medicines and Their Healing Properties*.

[45] Khare C. P. (2007). *Indian Medicinal Plants. An Illustrated Dictionary*.

[46] Kharat A. R., Nambiar V. V., Tarkasband Y. S., Pujari R. R. (2013). A review on phytochemical and pharmacological activity of genus *Ixora*. *International Journal of Research in Pharmacy and Chemistry*.

[47] Ching J., Chua T.-K., Chin L.-C. (2010). Beta-amyrin from *Ardisia elliptica* Thunb. is more potent than aspirin in inhibiting collagen-induced platelet aggregation. *Indian Journal of Experimental Biology*.

[48] United Nations (2010). *The Millennium Development Goals Report 2010*.

[49] World Health Organization (2007). *Recommendations for the Prevention of Postpartum Haemorrhage*.

[50] Aminimoghadamfarouj A., Nematollahi A., Wiart C. (2011). Anti-bacterial, antioxidant activity and phytochemical study of *Uvaria grandiflora*: a rare species of Annonaceae. *Journal of Pharmacy Research*.

[51] Rajeshwari E. (2012). Evaluation of anti-microbial activity of *Bambusa vulgaris* leaves. *International Journal of Phytotherapy Research*.

[52] Lagunday N. E., Cabana V. G. (2014). Taxonomy of ethnomedicinal botanicals and documentation of ethnomedicinal practices traditionally used by three selected ethnolinguistic communities in Mindanao, Philippines. *Asian Journal of Health*.

[53] Yeh L. L. L., Liu J.-Y., Lin K.-S. (2007). A randomised placebo-controlled trial of a traditional Chinese herbal formula in the treatment of primary dysmenorrhoea. *PLoS ONE*.

[54] So E. W. S., Ng E. H. Y., Wong Y. Y., Lau E. Y. L., Yeung W. S. B., Ho P. C. (2009). A randomized double blind comparison of real and placebo acupuncture in IVF treatment. *Human Reproduction*.

[55] Moerman D. E., Jonas W. B. (2002). Deconstructing the placebo effect and finding the meaning response. *Annals of Internal Medicine*.

[56] De Boer H. J., Lamxay V., Björk L. (2011). Steam sauna and mother roasting in Lao PDR: practices and chemical constituents of essential oils of plant species used in postpartum recovery. *BMC Complementary and Alternative Medicine*.

[57] Philippine Department of Health (2011). *MNCHN Manual of Operations*.

[58] Budzynska K., Gardner Z. E., Dugoua J.-J., Low Dog T., Gardiner P. (2012). Systematic review of breastfeeding and herbs. *Breastfeeding Medicine*.

[59] Anderson P. O., Valdés V. (2007). A critical review of pharmaceutical galactagogues. *Breastfeeding Medicine*.

[60] Bennett B. C. (2007). Doctrine of signatures: an explanation of medicinal plant discovery or dissemination of knowledge?. *Economic Botany*.

[61] Kwong T. C., Ryan R. M. (1997). Detection of intrauterine illicit drug exposure by newborn drug testing. *Clinical Chemistry*.

[62] Ahanya S. N., Lakshmanan J., Morgan B. L. G., Ross M. G. (2005). Meconium passage in utero: mechanisms, consequences, and management. *Obstetrical and Gynecological Survey*.

[63] Choi E.-M., Hwang J.-K. (2003). Investigations of anti-inflammatory and antinociceptive activities of *Piper cubeba*, *Physalis angulata* and *Rosa hybrida*. *Journal of Ethnopharmacology*.

[64] Bastos G. N. T., Santos A. R. S., Ferreira V. M. M. (2006). Antinociceptive effect of the aqueous extract obtained from roots of *Physalis angulata* L. on mice. *Journal of Ethnopharmacology*.

[65] Hulin A., Wavelet M., Desbordes J. M. (1988). Acute *Momordica charantia* (sorrossi) poisoning. Report of two cases. *Semaine des Hopitaux*.

[66] UNICEF (2014). *Levels and Trends in Child Mortality—Report 2014*.

